# Rearrangement of mitochondrial pyruvate dehydrogenase subunit dihydrolipoamide dehydrogenase protein–protein interactions by the MDM2 ligand nutlin‐3

**DOI:** 10.1002/pmic.201500501

**Published:** 2016-09-05

**Authors:** Luke Way, Jakub Faktor, Petra Dvorakova, Judith Nicholson, Borek Vojtesek, Duncan Graham, Kathryn L. Ball, Ted Hupp

**Affiliations:** ^1^ Institute of Genetics and Molecular MedicineUniversity of EdinburghEdinburghScotlandUK; ^2^ Regional Centre for Applied Molecular OncologyMasaryk Memorial Cancer InstituteBrnoCzech Republic; ^3^ CRUK & MRC Oxford Institute for Radiation OncologyUniversity of OxfordOxfordUK; ^4^ Centre for Molecular Nanometrology, WestCHEMDepartment of Pure and Applied ChemistryUniversity of StrathclydeGlasgowUK

**Keywords:** Cell biology, MDM2, Mitochondria, Nutlin‐3, P53, SWATH‐MS

## Abstract

Drugs targeting MDM2's hydrophobic pocket activate p53. However, these agents act allosterically and have agonist effects on MDM2's protein interaction landscape. Dominant p53‐independent MDM2‐drug responsive‐binding proteins have not been stratified. We used as a variable the differential expression of MDM2 protein as a function of cell density to identify Nutlin‐3 responsive MDM2‐binding proteins that are perturbed independent of cell density using SWATH‐MS. Dihydrolipoamide dehydrogenase, the E3 subunit of the mitochondrial pyruvate dehydrogenase complex, was one of two Nutlin‐3 perturbed proteins identified fours hour posttreatment at two cell densities. Immunoblotting confirmed that dihydrolipoamide dehydrogenase was induced by Nutlin‐3. Depletion of MDM2 using siRNA also elevated dihydrolipoamide dehydrogenase in Nutlin‐3 treated cells. Mitotracker confirmed that Nutlin‐3 inhibits mitochondrial activity. Enrichment of mitochondria using TOM22+ immunobeads and TMT labeling defined key changes in the mitochondrial proteome after Nutlin‐3 treatment. Proximity ligation identified rearrangements of cellular protein–protein complexes in situ. In response to Nutlin‐3, a reduction of dihydrolipoamide dehydrogenase/dihydrolipoamide acetyltransferase protein complexes highlighted a disruption of the pyruvate dehydrogenase complex. This coincides with an increase in MDM2/dihydrolipoamide dehydrogenase complexes in the nucleus that was further enhanced by the nuclear export inhibitor Leptomycin B. The data suggest one therapeutic impact of MDM2 drugs might be on the early perturbation of specific protein–protein interactions within the mitochondria. This methodology forms a blueprint for biomarker discovery that can identify rearrangements of MDM2 protein–protein complexes in drug‐treated cells.

**Abbreviations**
CES: collision energy spread
FA: formic acid
FASP: filter‐aided sample preparation
TIC: total ion current
Wt: wild‐type

## 1 Introduction

The ubiquitin conjugation system has emerged as an extensive landscape of untapped potential for drug discovery 1. Ubiquitination can impact on protein turnover, specific‐activity, and trafficking 2. Ubiquitin attachment requires the concerted action of an E1, initiator ubiquitin‐activating enzyme; an E2, ubiquitin‐conjugating enzyme; and an E3, ubiquitin‐ligase adaptor that mediates ubiquitin transfer onto a substrate 3. The E3 ligases can be divided into HECT or RING domain (which includes U‐Box and PHD) containing proteins. The proto‐oncogene, MDM2, is a RING‐domain containing E3‐ubiquitin ligase that regulates the function of the p53 tumor suppressor 4. The dominant role of MDM2 as an inhibitor of p53 has led to the identification of small molecules that bind to the N‐terminal hydrophobic cleft of MDM2 leading to the activation of p53 transcriptional activity 5.

**Significance of the study**

Reactivating the p53 tumor suppressor forms a central strategy in anti‐cancer therapeutics. Drugs are being developed that target a key inhibitor of p53, named MDM2. In cell lines and in the clinic, MDM2‐targeted drugs can have partial agonist effects. Identifying dominant non‐p53 targets of these MDM2‐targeted drugs would improve patient stratification. In this study, we use SWATH‐based MS to identify the most dominant target that responds at an early time after drug treatment. We identified mitochondrial proteins and the pyruvate dehydrogenase complex in particular as a selective target of a MDM2‐targeted drug. This methodology forms a blueprint for biomarker discovery that can identify rearrangements of MDM2 protein–protein complexes in drug‐treated cells.

With the compelling scientific case for reactivating the wild‐type (wt)‐p53 pathway by targeting MDM2, there are numerous MDM2 ligands in preclinical or clinical trials 1. Human sarcomas often exhibit *mdm2* gene amplification 6 7 and such patient groups provide a test‐bed for MDM2 targeted therapeutics. Indeed, small molecules have recently been evaluated in clinical trials in *mdm2*‐amplified sarcomas with partial success; more than half of patents exhibiting “stable” disease 8. The lack of tumor regression in the majority of patients in this trial appears to be due to “on‐target toxicity” or an “agonist property” with this class of MDM2 drug 9. For example, Nutlin‐3 can stabilize the oncoprotein Notch 10 and it follows that the balance of stabilization of tumor suppressor or oncoproteins can tip the balance of tumor regression. Non‐p53 companion biomarkers are required to identify MDM2‐drug responsive or resistant patients 11. This is consistent with recent data suggesting that MDM2 has emerging oncogenic roles and “druggable” functions that are independent of p53 12, 13, 14. So one key question we need to ask to improve patient response to MDM2 directed drugs is by what mechanisms could the Nutlins promote an “on‐target toxicity”?

The Nutlin family of molecules bind to the hydrophobic pocket of MDM2 mimicking its inhibitory interaction with the transactivation domain of p53 15. This activates p53 transcription by dissociating the p53‐MDM2 complex 5. Nutlin does not however inhibit MDM2 E3‐ligase activity 16. In fact research has shown that Nutlin functions as an allosteric agonist promoting a lower affinity, but physiologically significant, interaction between the core DNA‐binding domain of p53 and the central acidic domain of MDM2 16, 17. It is this second interaction between p53 and its oncogenic E3‐ligase partner that constitutes an “ubiquitination signal” for p53 ubiquitination. More recently, the prooncogenic protein Notch has been shown to be monoubiquitinated and activated by MDM2 using the same “dual‐site” mechanism as MDM2 uses for p53 10. Nutlin can also promote the deoligomerization of NPM 18 providing additional evidence for an agonist effect of Nutlin on MDM2 protein–protein interactions. The Nutlin‐3 responsive proteins can be stratified with respect to p53‐like “BOX‐I” homology motifs that identify a relatively large set of p53‐like MDM2‐binding proteins whose equilibrium binding to MDM2 is disturbed by Nutlin‐3 11.

Based on the information introduced above, cell‐based quantitative proteomics screens were set up that aimed to identify dominant subcellular organelles that are affected relatively early after Nutlin‐3 treatment and that could explain its “on target toxicity” [9]. Our hypothesis is focused on the concept that Nutlin‐3 can promote changes in the MDM2‐interactome by either dissociating existing PPIs or induction of new MDM2 interactions. Our experimental design included cells grown at two different densities and treated with Nutlin‐3, since cell density changes can alter MDM2 steady‐state levels 19 and cell density can impact on p53 activity 20. We identified mitochondrial proteins as a group of proteins responding early to Nutlin‐3 treatment and have validated key changes in the protein–protein interaction partners of one mitochondrial protein within the pyruvate dehydrogenase complex. These data highlight the pronounced effect Nutlin‐3 has on the integrity of mitochondrial proteome and is consistent with a previous report that identified mitochondrial perturbation as a key effect of Nutlin‐3 21. This proteomics platform can facilitate defining biomarkers of Nutlin‐3 that mediate drug response or resistance using clinical samples in the future.

## 2 Materials and methods

### 2.1 Cell culture

HCT116 cells were grown in McCoys 5A Medium including 10% fetal bovine serum at 37˚C with 5% CO_2_. Treatment of cells with either 0.05% DMSO or 20 μM Nutlin‐3 (Enzo Life Sciences) was executed 4, 8, or 24 h before an experiment, as indicated in the figure legends. MitoTracker Red CMXros (100 nM) and Leptomycin B (40 nM) were added in proximity ligation experiments 45 min prior to fixing. For Western blotting cells were either scraped at 40% cell density (lower confluence) or at 80% cell density (higher confluence) for experimental use. Following washing with ice cold PBS, the cells were lysed in Urea lysis buffer (8 M Urea, 25 mM HEPES (pH7.5), 25 mM NaCl, 0.05% Triton x‐100, and 5 mM DTT) for 15 min on ice, centrifuged at 13 000 rpm for 15 min and the supernatant saved. Protein concentrations were determined by the Bradford method 22. Samples for MS (either SWATH or Tandem Mass Tag [TMT] label) were scraped and lysed using UA buffer (8 M Urea, 0.1 M Tris‐Cl (pH 8), and a protease inhibitor mixture; Calbiochem; 539134), at approximately 5 cell‐pellet volumes. Following pellet resuspension, lysates were snap frozen, thawed, sonicated (Sonics, Vibra‐Cell™, Sonics & materials, Danbury, USA), and then centrifuged at 14 000 rpm for 30 min at 4˚C. The supernatant was saved and protein concentration was determined by BCA (according to manufacturer's protocols; Thermo fisher; kit code 23225). For siRNA treatment, A375 cells were transfected with 50 nm MDM2 SMARTpool siRNA (Dharmacon #M‐003279‐04‐0010) using DharmaFECT‐4 reagent and incubated for 24 h. Cells were then treated with either 0.05% DMSO or 20 μM Nutlin‐3 for 4 h before harvesting and lysing using urea lysis buffer. Nontargeting siRNA (Dharmacon 001810‐10‐05) was used in parallel as a control.

### 2.2 Protein purification

wt‐dihydrolipoamide dehydrogenase and wt‐MDM2 were cloned into pDEST17 vector, containing glutathione S‐transferase N‐terminal tag, expressed into BL21 (DE3) competent *E. coli* cells and grown O/N. The dihydrolipoamide dehydrogenase gene was obtained from Origene (RG200639 DLD (GFP‐tagged)—Human dihydrolipoamide dehydrogenase). The cells were subcultured and induced with 1 M IPTG after OD 0.4 had been reached. After 3 h the cells were pelleted and incubated with lysis buffer (10% sucrose, 50 mM Tris (pH 8), 150 mM NaCl, 3 mg Lysozyme, 0.5% NP40, 5 mM DTT, 1 mM Benzamidine, 20 μg/mL leupeptin, 1 μg/mL aprotinin, 2 μg/mL pepstatin, 10 μg/mL soybean trypsin inhibitor, 1 mM EDTA) for 45 min before a 1‐min incubation at 37˚C and sonication on ice. The lysates were then centrifuged at 4000 rpm for 15 min and the supernatant added to glutathione sepharose 4B beads and incubated for 2 h at 4˚C. The beads were washed using wash buffer (20 mM HEPES (pH 7.5), 1 mM DTT, 10% glycerol, 150 mM NaCl) before elution buffer (25 mM HEPES (pH 7.5), 1 mM DTT, 10% glycerol, 150 mM NaCl) containing PreScission protease. Purification of proteins was accessed using Coomassie staining and Western blotting. P53 was purified according to prior methods 23. The crude lysate derived from *E. coli* cells overproducing p53 from T7.7 promoter after IPTG induction (Fraction I) was diluted fivefold in Buffer B (25 mm HEPES (pH8.0), 0.1 mM EDTA, 1 mM Benzamidine, 1 mM DTT) and applied to a Hi‐Trap Heparin‐Sepharose column. Bound protein was eluted with a 10 column volume linear gradient in Buffer E from 0.1–1 M KCL. Fractions of p53 were identified by ELISA, were pooled, (Fraction II), diluted fivefold with Buffer B, and applied to a cellulose‐phosphate column. Bound protein was eluted with a 20 column volume linear gradient in Buffer B from 0.1–1 M KCL.

### 2.3 Protein–protein interaction assays

Polystyrene solid‐phase microtiter wells (Costar; 07‐200‐336) were coated with 100 ng of purified wt‐dihydrolipoamide dehydrogenase or wt‐p53 in 0.1 M NaHCO_3_ (pH 8.6). The plate was blocked with 3% BSA/0.1% PBS‐Tween‐20 after which a titration of wt‐MDM2, that had been incubated with either 0.05% DMSO or 20 μM Nutlin‐3 for 15 min at RT, was incubated in the wells for 1 h at RT. Following washing in 0.1% PBS‐Tween‐20 2A10 mAb (1:1000) was used to detect MDM2, the plate washed and incubated with HRP‐tagged rabbit anti‐mouse secondary antibody (1:2000). After final washes with 0.1% PBS‐Tween‐20, ECL solution was added and the Ascent Fluoroskan plate reader at 450 nm used to quantify the experiment.

### 2.4 Proximity ligation and Immunofluorescence assays

HCT116 cells were grown in six‐well plates over glass coverslips (16 mm diameter) until 50% confluency was achieved with cells treated with 0.05% DMSO or 20 μM Nutlin‐3 as described earlier. The cells were fixed onto slides with 4% paraformaldehyde in PBS for 20 min at RT, permeabilized for 10 min in 0.25% Triton x‐100 in PBS and blocked with 3% BSA in PBS for 30 min. Antibodies from different species were then incubated on the slides, with combinations of MDM2 mouse mAb (4B2) with either rabbit pAb DLD (sc‐135027; Santa Cruz) or p53 (CM‐1), at a 1:250 dilution for 1 h at RT. Following PBS washes IF coverslips were incubated with either 594 nm goat anti‐mouse or 488 nm Donkey anti‐rabbit antibodies for 1 h at RT. IF coverslips were further washed in PBS stained with DAPI and mounted onto slides with fluorescent mounting medium. Proximity ligation assay (PLA) was carried out with the OLIGO duolink 24 designated protocol using anti‐mouse and anti‐rabbit probes (Sigma; The duolink probe product numbers are 92002 (rabbit plus), 92004 (mouse minus), and the duolink green detection is 92014.) The PLA coverslips were stained with DAPI and mounted in the same fashion as the IF coverslips. Images were taken at 40X using an Olympus BX51 microscope.

### 2.5 Sample preparation for MS

Samples were trypsinized according to previously published methods for FASP (filter‐assisted sample preparation) digestion protocols 25. The filter unit (Hydrosart—stabilized cellulose‐based membrane; Vivacon 500 mL (Sartorius Stedim Biotech, Germany); product number is: VN01H02 with a 10 kDa cutoff). The filter unit was washed with 100 μL of a buffer containing 8 M Urea, 0.1 M Tris/HCl pH 8.5 (UA) centrifuged at 14 000 rpm/15 min (room temperature). Two hundred microliters of UA buffer and 100 μg of the protein sample in urea lysis buffer was added to the filter unit followed by centrifugation for at 14 000 rpm/15 min (room temperature). This step results in proteins absorbing to the matrix and removal of the lysis buffer components. UA buffer containing 16.7 mM TCEP was then added followed by incubation in a thermomixer for 600 rpm/30min (37˚C) followed by centrifugation for 14 000 rpm/15 min (room temperature). This step results in reduction of any disulfide bonds in the denatured proteins. The filter unit was then incubated with UA containing 50 mM iodoacetamide in the dark for 20 min at room temperature before further centrifugation at 14 000 rpm/15 min. This step catalyzes alkylation of free sulfhydryl groups to form S‐carboxyamidomethyl‐cysteine that cannot be reoxidized thus permitting maximal tryptic cleavage. The filter unit was washed with a buffer containing 100 mM NH_4_HCO_3_, centrifuged at 14 000 rpm/15 min (room temperature). A buffer containing 50 mM NH_4_HCO_3_ (100 μL) and trypsin in a mass ratio 1:30 (trypsin/protein) was added to the filter, the samples were mixed at 600 rpm/1 min, and then incubated for 18 h in a wet chamber (37˚C). Two rounds of subsequent centrifugation 14 000 rpm/15 min (room temperature) eluted the peptides. The peptide purification of samples followed with the evaporated samples being resuspended in 0.1% formic acid (FA). Micro SpinColumns C‐18 (25–75 μL) (Harvard Apparatus, USA) were conditioned twice with 100% ACN/0.1% FA, before washing with 0.1% FA. The column was hydrated for 15 min in 0.1% FA, centrifuged and the sample peptides added. After centrifugation (500 rpm/2 min), peptides were eluted from the column using three buffers, first with 50% ACN and 0.1% FA in water, second in 80% can and 0.1% FA in water and third composed from 0.1% FA in ACN. Subsequently, the samples were evaporated using a SpeedVac.

### 2.6 SWATH‐MS

SWATH method for label‐free quantification of proteins in complex mixtures was set‐up according to previously published methods 26. TripleTOF 5600+ (AB‐SCIEX, Toronto, Canada) operated in high sensitivity positive mode. Random precursor ion peaks were extracted from TOF‐MS and the approximate chromatographic peak width was defined to correctly establish SWATH method so that at least ten data points were acquired across a peak. Four randomly extracted precursor peaks from TOF‐MS were evaluated and the peak width at FWHM was in average 1.5 min, so the cycle time of SWATH was set to 3.5 s. With the defined cycle time an optimal SWATH width of 20 Da with 1 Da overlap was calculated, with accumulation time 98 ms per SWATH. Precursor range was selected from 400 amu up to 1100 amu. Product ion range was scanned from 300 amu up to 1600 amu and rolling collision energy was used with collision energy spread (CES) of 10 mV. Spectral library for SWATH data mining was measured from 1 μL pool of cell lysates (approx. 1 μg/μL protein concentration). Mass spectrometer TripleTOF 5600+ (AB‐SCIEX, Toronto, Canada) operated in data‐dependent mode. During each cycle, mass spectrometer fragmented the top 20 intense precursor ions with exclusion time set to 12 s. Minimum precursor ion intensity was set to 50 cps, 100 ms accumulation time was used and 150 ms accumulation time for TOF‐MS scan. For building up of spectral library 1632 proteins FDR 1% were used after Protein Pilot 4.5 (AB‐SCIEX, Toronto, Canada) search using Uniprot 2013_12 database. Spectral library was built in Peakview software 1.2.0.3 (AB‐SCIEX, Toronto, Canada), only the identifications below FDR of 1% were indexed. Quantitative data (peak areas) corresponding to each protein included in spectral library were extracted from SWATH data using manual analysis in Peak view 1.2.0.3 (AB‐SCIEX, Toronto, Canada). Data were extracted using retention time window of 3.5 min, which was determined by extracting random peaks across LC gradient (Fig. 2A and B). Retention time window describes the LC retention time shifts between SWATH technical replicates and data dependent acquisition (DDA) measurement and specifies in which scope of retention times software should look for peaks included in spectral library (DDA measurement result). Eight peptides per protein and five product ions per each peptide were used. Extracted quantitative data were further analyzed in Marker view where T‐testing was done on quantitative data from all replicates originating from compared sample pair. As a result, for all proteins in spectral library protein fold changes and *p* values (in Supporting Information Tables 1 and 2) between chosen sample pair were calculated and are valid only for the concrete pair comparison.

### 2.7 Isobaric (TMT) labeling of TOM22+ antibody‐enriched mitochondrial proteins

Mitochondrial isolation and lysis was performed according to the protocols defined by the Miltenyl Biotec mitochondrial isolation kit (product code 130‐094‐532). Cells processed in biological duplicates were lysed in urea buffer (100 μL of 8 M urea in 0.1 M Tris‐Cl, pH 8) containing protease inhibitors (1:100; as above). Samples were mixed by pipetting and stored overnight in the fridge, then were sonicated and centrifuged at 14 000 rpm at 4°C for 30 min. The proteins concentration was determined by the RCDC kit (Bio Rad; 5000121). Proteins were digested into peptides with the FASP protocol 25 and detailed as above. Fifty micrograms of peptides per sample were labeled with TMT labels, according to Thermo Fisher Scientific (TMT 10plex™ Label Reagent Set, Prod # 90111, Lot# QB213026). The tags, the mass, and the sample connections are summarized in Fig. 5A. Peptides were separated using Thermo Scientific UltiMate 3000 RSLCnano chromatograph. Sample was loaded on a precolumn (Thermo Scientific μ‐precolumn, 30 μm id, 5 mm length, C18 PepMap 100, 5 μm particle size, 100 Å pore size) and peptides further separated on a Thermo Scientific^TM^ Acclaim PepMap RSLC column (75 μm id, length 500 mm, C18, particle size 2 μm, pore size 100 Å) using a 300 nL/min flow rate with a linear gradient of B (80% ACN in 0.08% aq. formic acid) in A (0.1% aq. formic acid). The gradient composition used for peptides separation was as follows highlighting time as a function of %B: 0 min, 2%B; 10 min, 2%B, 200 min, 50%B, 210 min, 98%B, 228 min, 98%B, 255 min END. Peptides eluting from the column were introduced into Thermo Scientific^TM^ Orbitrap Elite^TM^ operating in Top10 data‐dependent acquisition mode. The data acquisition parameters setting for the Top10 method include: MS1 includes a mass range of 335–1800, a resolving power setting of 120 000, and a Max. in time of 200 ms; MS2 includes: HCD activation type; a min. signal required of 5000, an isolation width of 1.2, NCE of 35, charge rejection state is unassigned; 1+; dynamic exclusion settings are ON; the exclusion width is 10 ppm; repeat count is set at 1; the exclusion list size is 500; and the exclusion duration is 30 s.

### 2.8 Database searching and analysis

The data were processed with a Proteome Discoverer1.4 (Thermo Scientific^TM^), employing Mascot with the following search settings: database Swiss‐Prot human (April 2015); enzyme trypsin; two missed cleavage sites; precursor mass tolerance 10 ppm; fragment mass tolerance 0.6 Da; dynamic modifications: formyl [peptide N‐terminus], oxidation [M], Gln to pyro‐Glu [peptide N‐terminus], acetyl [protein N‐terminus], TMT6plex [K], TMT6plex [N‐term], and static modification: carbamidomethyl [C]. The results of the search were further submitted to generate the final report using a cutoff of 1% FDR on peptide levels and only unique peptides were used for protein quantitation. The quantitative option was enabled with the corresponding combination of labeled peptides in the sample type and the observed relative quantification ratio was normalized compared to the median. Proteins with a fold change of 2.0 were considered as highly differentially expressed (Fig. 5C) and using a less stringent cut‐off of 1.5‐fold changes we identified a larger list of mitochondrial proteins (Table 1). MS/MS^ALL^ SWATH data and MS/MS data from TMT experiment are uploaded in PRIDE.

**Table 1:** Data summarize the proteins showing the highest fold change (elevated or reduced peptide IDs) after Nutlin‐3 treatment in the mitochondrial immunoprecipitation using TMT

Accession	Gene name	Protein name	Mitochondrial?	Fold change	
				Nutlin/DMSO 1	Nutlin/DMSO 2
*A. Nutlin‐3 upregulated proteins in TOM22+ mitochondria*					
O60814	H2B1J	Histone H2B type 1‐J	No	3.75	2.19
P06899	H2B1K	Histone H2B type 1‐K	No	3.69	1.88
P14927	NDUA2	NADH dehydrogenase 1 alpha subcomplex subunit 2	Yes	2.03	2.40
P51970	QCR7	Cytochrome b‐c1 complex subunit 7	Yes	1.91	1.96
P52815	NH2L1	NHP2‐like protein 1	No	1.78	1.84
P55769	RUVB2	RuvB‐like 2	No	1.66	1.63
Q9Y230	RM12	39S ribosomal protein L12	Yes	1.56	1.90
O43678	NDUA8	NADH dehydrogenase 1 alpha subcomplex subunit 8	Yes	1.52	1.98
*B. Nutlin‐3 downregulated proteins in TOM22+ mitochondria*					
P62805	HIST1H4A	Histone H4	No	0.09	0.12
Q8NDV3	SMC1B	Structural maintenance of chromosomes protein 1B	No	0.14	0.46
Q9UJF2	RASAL2	Ras GTPase‐activating protein nGAP	No	0.18	0.40
Q6SA08	TSSK4	Testis‐specific serine/threonine‐protein kinase 4	No	0.20	0.47
Q9BPU6	DPYSL5	Dihydropyrimidinase‐related protein 5	No	0.21	0.36
P01008	SERPINC1	Antithrombin‐III	No	0.22	0.31
Q8IUG5	MYO18B	Unconventional myosin‐XVIIIb	No	0.24	0.50
P02768	ALB	Serum albumin	No	0.24	0.42
P05141	SLC25A5	ADP/ATP translocase 2	Yes	0.28	0.44
Q03181	PPARD	Peroxisome proliferator‐activated receptor delta	No	0.28	0.64
P12236	SLC25A6	ADP/ATP translocase 3	Yes	0.32	0.39
Q00325	SLC25A3	Phosphate carrier protein	Yes	0.32	0.49
O95202	LETM1	LETM1 and EF‐hand domain‐containing protein 1	Yes	0.34	0.26
P13645	KRT10	Keratin type I cytoskeletal 10	No	0.34	0.28
P35908	KRT2	Keratin type II cytoskeletal 2 epidermal	No	0.35	0.37
Q6NXT2	H3F3C	Histone H3.3C	No	0.36	0.27
P35527	KRT9	Keratin type I cytoskeletal 9	No	0.37	0.21
P10412	HIST1H1E	Histone H1.4	No	0.39	0.37
P04264	KRT1	Keratin type II cytoskeletal 1	No	0.40	0.25
Q04837	SSBP1	Single‐stranded DNA‐binding protein	Yes	0.41	0.67
P07900	HSP90AA1	Heat shock protein HSP 90‐alpha	No	0.43	0.62
P00403	MT‐CO2	Cytochrome c oxidase subunit 2	Yes	0.43	0.61
P27824	CANX	Calnexin	Yes	0.43	0.36
P19338	NCL	Nucleolin	No	0.47	0.60
O00217	NDUFS8	NADH dehydrogenase iron‐sulfur protein 8	Yes	0.52	0.65
P68104	EEF1A1	Elongation factor 1‐alpha 1	No	0.54	0.63
P08238	HSP90AB1	Heat shock protein HSP 90‐beta	No	0.57	0.52
P02656	APOC3	Apolipoprotein C‐III	No	0.58	0.30
O43707	ACTN4	Alpha‐actinin‐4	No	0.59	0.58
Q8NEY8	PPHLN1	Periphilin‐1	No	0.60	0.49
P56537	EIF6	Eukaryotic translation initiation factor 6	No	0.61	0.65
Q8IY81	FTSJ3	pre‐rRNA processing protein FTSJ3	No	0.66	0.42

The raw data (Supporting Information Tables 3 and 4) were normalized over the internal average for each replicate. The *p* value using a paired *t*‐test is 0.269194 indicating that the two datasets are not significantly different. The samples are highlighted with respect to whether or not they are classically known to be mitochondrial proteins (shaded) with the caveat that some proteins, like histones, have been recently reported to interact with mitochondria 45, 46, 47.

## 3 Results

### 3.1 Designing an experimental plan to identify novel MDM2 drug‐responsive‐binding proteins

**Figure 1:**
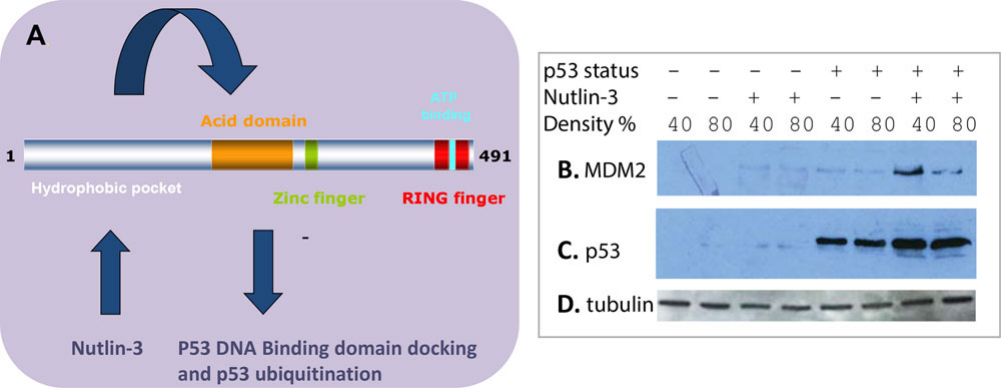
The allosteric effect of Nutlin‐3 on MDM2 functions. (A) MDM2 has multiple functional domains including an N‐terminal peptide‐binding groove that is the binding site of Nutlin‐3 5; a central domain that responds allosterically to N‐terminal MDM2‐binding ligands (like Nutlin‐3) to increase p53 binding and ubiquitination (arrows) 16, and a RING domain that is required for E2 docking 49 and allosteric control of ubiquitination by the E2, UBCH5 19. N‐terminal domain Nutlin‐3 binding by MDM2 can stimulate p53 ubiquitination 16, stimulate ubiquitin‐dependent protein activation of Notch 10, and direct binding to alter target protein oligomerization of NPM 50. Thus, Nutlin‐3 can dissociate or induce various protein–protein interactions due to the allosteric effects of ligands on MDM2 function. These data suggest a complex effect of MDM2 ligands on changes in the steady‐state cellular proteome. (B–D) HCT116 cells (p53+ and p53‐null, as indicated) where grown to 40 and 80% density as defined in the methods. Cells were treated with Nutlin‐3 (20 μM final concentration) or DMSO control and after 4 h cells were harvested for lysis without proteasome inhibitor treatment (that would artificially elevate levels of target protein). Lysates were blotted with the indicated antibodies (MDM2, p53, and tubulin as a loading control). The data demonstrate that p53 protein is induced equivalently by Nutlin‐3 at either cell density, but MDM2 protein exhibits higher steady‐state levels at the lower density after Nutlin‐3 treatment, as reported previously 19.

There are over one‐hundred published MDM2‐binding proteins that have been discovered using a large range of cell types, methodologies, and experimental conditions 27. This vast number of MDM2‐interactors has not been integrated into MDM2 “signaling pathways.” These target proteins could act as biomarkers to predict MDM2 drug responses but identifying such “core” MDM2‐binding proteins using tissue culture approaches is complicated by the fact that cell density can have a significant effect on p53 protein synthesis [28] and on the steady‐state levels of MDM2 protein through phosphorylation of its pseudo‐substrate motif 19, 28. In addition, cell density changes created by changing the cell number at time of seeding also can attenuate p53‐dependent biological processes; this effect of cell density is not a consequence of density‐dependent cell‐cycle changes but rather are linked to cadherin‐mediated cell‐cell junctions 20. Thus, it is likely that density effects on MDM2 levels will alter its steady‐state‐binding proteins that in turn impacts on the balance between oncoprotein or tumor suppressor protein stabilization. Such a density effect might explain in part the striking heterogeneity of p53 protein stabilization in primary human tumors in cancer tissue [29]. Our experimental design in this current study aimed to use the same wt‐p53 containing cancer cell line plated at two different cell numbers that show differential MDM2 induction, but similar p53 induction, by Nutlin‐3 (Fig. 1). The use of the same cell line plated at two different densities would allow us to subtract any density‐specific contributions to MDM2 drug responses. This would produce a very stringent screen to identify only those proteins that commonly change upon Nutlin‐3 treatment independent of MDM2 protein differential stabilization (Fig. 1A).

### 3.2 Identification of the most significantly perturbed proteins induced by the MDM2 ligand Nutlin‐3 at two cell densities that reflect differential MDM2 protein induction

We defined the parameters that measured differential MDM2 protein levels as a function of differences in the cell number at the time of cell plating. The treatment of HCT116 cells (p53+) with Nutlin‐3 at 40 or 80% plating density resulted in higher MDM2 protein induction at lower compared to higher cell plating density (Fig. 1B–D, lanes 7 versus 8). This is consistent with previous data showing that either endogenous or transfected MDM2 protein has lower steady‐state levels at higher cell density 19, 28.

**Figure 2:**
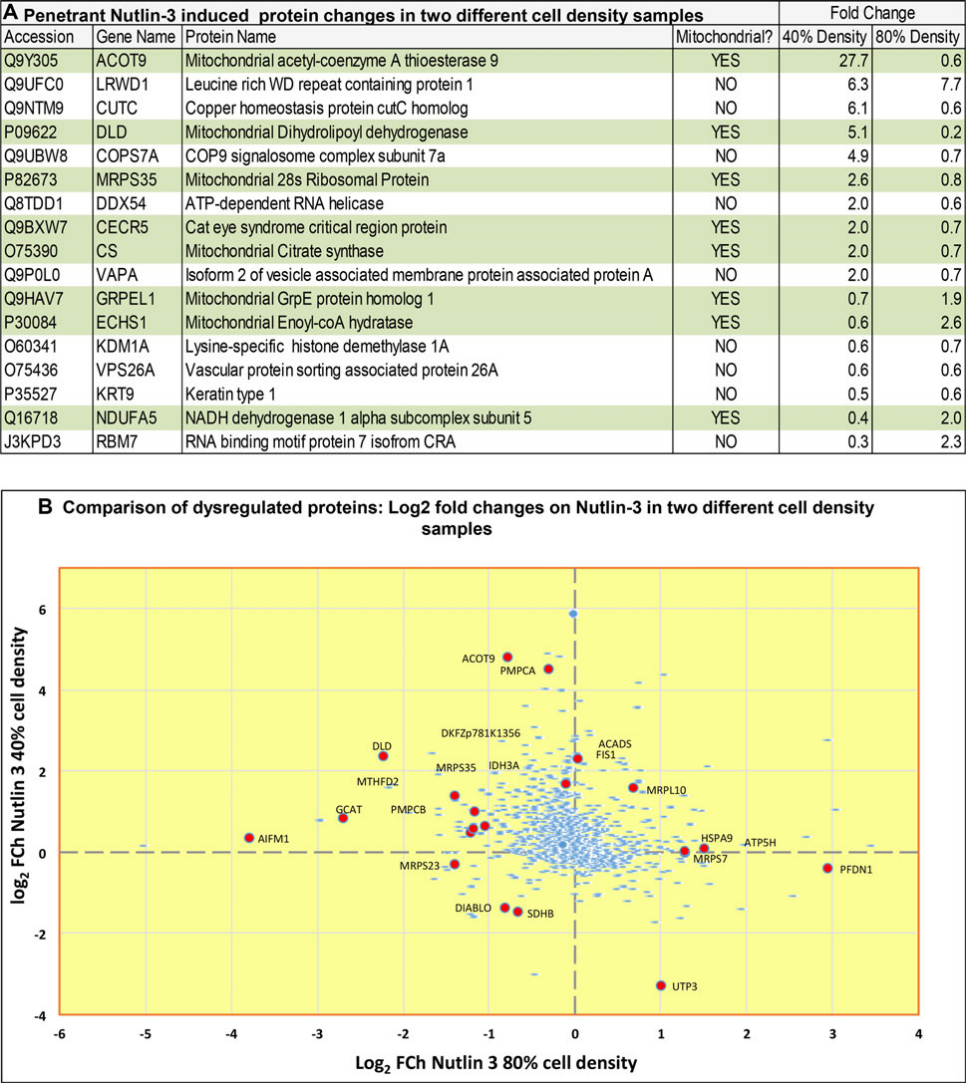
Identification of proteins with the most differential change at 40 and 80% cell density in response to Nutlin‐3 treatment. (A) Identification of proteins perturbed by Nutlin‐3 using SWATH MS. HCT116 cells were incubated for 4 h with Nutlin‐3 under conditions in which MDM2 is just beginning to be stabilized (Fig. 1B). This ensures that we capture changes in the cellular proteome just when MDM2 is starting to be perturbed in cells by Nutlin‐3. The cell pellets were processed using MS SWATH 26 to identify differentially perturbed proteins (Supporting Information Tables 1 and 2), some of which are highlighted (in green) as a function of 40 or 80% cell density. (B) A scatter plot of the total protein changes as a function of cell density and fold change (log_2_) with mitochondrial proteins highlighted in red.

We aimed to identify proteins whose levels were affected most significantly by Nutlin‐3 independent of cell plating density (e.g. MDM2 protein levels) and then to link these targets to changes in key cellular phenotypes. HCT116 (p53+ and isogenic p53‐null) cells were grown in parallel to 40 or 80% density and treated with DMSO control or Nutlin‐3 for 4 h. The use of the same cell line at two different densities where MDM2 protein levels are differential stabilized provides a subtraction of density‐specific contributions to MDM2 protein stabilization. Cells from two densities were then harvested and subjected to differential protein quantitation using label‐free methodologies (SWATH‐MS 26; Supporting Information Fig. 1A and B; Supporting Information Tables 1 and 2). It is important to point out that SWATH analysis involved the application of three technical replicates of each sample, but that there are no biological replicates of each sample. This precludes us developing statistically significant pathway maps at two cell densities, but it allows us to identify outlier responders that require orthogonal validation for confirmation. The four‐hour time point was used since this precedes major visible changes in cell phenotype and is the time point previously shown to begin to reveal changes in dozens of proteins 18. Both samples were processed in triplicate; for example, the total ion current (TIC) from three technical replicates of Nutlin‐3 p53+ sample from 40% density is shown in Supporting Information Fig. 1A and B. This defines the accuracy of the autosampler sample pickup and reproducibility of sample loading. The numbers of proteins identified at 40% cell density were 1540 and those at 80% cell density were 1535 (Supporting Information Tables 1 and 2). Proteins that changed 2‐fold or greater as defined by SWATH‐MS in response to Nutlin‐3 (increased or decreased peptide ion quantitation) at both low and high cell densities were tabulated (Fig. 2A). These data are depicted in a scatter plot of total protein changes (as defined by increased or decreased peptide ion quantitation) as a function of both densities (e.g. biological replicates) (Fig. 2B). Only two proteins changed by more than 2 x log_2_ at both densities (Fig. 2B); those being the mitochondrial protein dihydrolipoamide dehydrogenase 30 and the nucleosome interacting protein LRWD1 [31]. Additional mitochondrial protein changes (increased or decreased peptide ion quantitation) were also identified by Nutlin‐3 such as NADH dehydrogenase subunit 5, the chaperone GRPEL1, mitochondrial 28S ribosomal protein MRPS35, mitochondrial acyl‐coenzyme A thioesterase 9, citrate synthase, and mitochondrial enoyl‐coA hydratase (Fig. 2A and B; Supporting Information Tables 1 and 2). As samples were not processed as biological replicates, this precludes the formation of statistically significant “pathway maps.” As such, we focused on validation of individual outliers as potential core MDM2 interactors.

**Figure 3:**
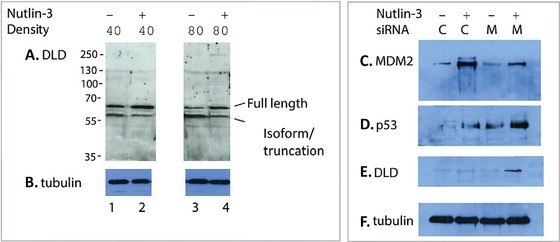
Immunochemical analysis of dihydrolipoamide dehydrogenase protein levels after MDM2 perturbation. (A and B) HCT116 cells (p53+) where grown to 40 or 80% density. Cells were treated with Nutlin‐3 (20 μM final concentration) or DMSO control and after 4 h, cells were harvested for lysis without proteasome inhibitor treatment that would artificially elevate levels of target protein. Lysates were blotted with the indicated antibodies to dihydrolipoamide dehydrogenase and tubulin as a loading control. (C–F). HCT116 cells grown at 80% density and then were treated with control siRNA (C) or siRNA to deplete MDM2 (M) for 24 h, followed by treatment with DMSO or Nutlin‐3. The lysates were then immunoblotted for p53, MDM2, and dihydrolipoamide dehydrogenase, and the loading control.

Dihydrolipoamide dehydrogenase was evaluated immunochemically and at either cell density two effects were observed; the full‐length isoform of dihydrolipoamide dehydrogenase was induced by Nutin‐3 and the lower isoform of dihydrolipoamide dehydrogenase was reduced by Nutlin‐3 (Fig. 3A). It is important to note that, although fold changes in peptide ions identified from dihydrolipoamide dehydrogenase (and other proteins; Supporting Information Tables 1 and 2) can be used to define protein changes as being “up or downregulated” at the different densities, this does not necessarily reflect changes in absolute steady‐state protein levels. It might reflect the extractability, PTM, and trypsinization capacity that give rise to peptide ion identification and then quantitation using MS. For example, tryptic dihydrolipoamide dehydrogenase peptide ions are elevated by Nutlin‐3 at 40% cell density, but are suppressed at 80% cell density (Fig. 2A and B). However, total dihydrolipoamide dehydrogenase protein upper isoform is elevated by Nutlin‐3 at both cell densities and the lower isoform is suppressed at both cell densities as defined by immunoblotting. Different buffers are used for immunoblotting and sample processing for SWATH‐MS. Nevertheless, these data highlight that the mitochondrial protein dihydrolipoamide dehydrogenase protein is perturbed by Nutlin‐3. Given the prior report that Nutlin‐3 promotes the generation of mitochondrial reactive oxygen species and p53 protein translocation 21, 32, we focused in this study on evaluating mitochondrial proteome changes when MDM2 is perturbed at early time points after Nutlin‐3 treatment and whether this is linked to novel MDM2‐mitochondrial‐binding proteins. We also employed siRNA as an approach to target MDM2 and examine effects on dihydrolipoamide dehydrogenase protein levels. siRNA is complicated to use since it also activates the interferon response 33. In addition, siRNA depletion of MDM2 is known to activate p53 (Fig. 3B), which in turn induces MDM2 protein so that MDM2 levels are only attenuated by siRNA (Fig. 3B). Nevertheless, this treatment can induce the upper isoform of dihydrolipoamide dehydrogenase (Fig. 3B). These data support the idea that “inhibiting” MDM2 with the ligand Nutlin‐3 or with siRNA (that both stabilize p53 protein) can induce dihydrolipoamide dehydrogenase.

### 3.3 Evaluation of global effects of Nutlin‐3 on the mitochondrial proteome and respiration

**Figure 4:**
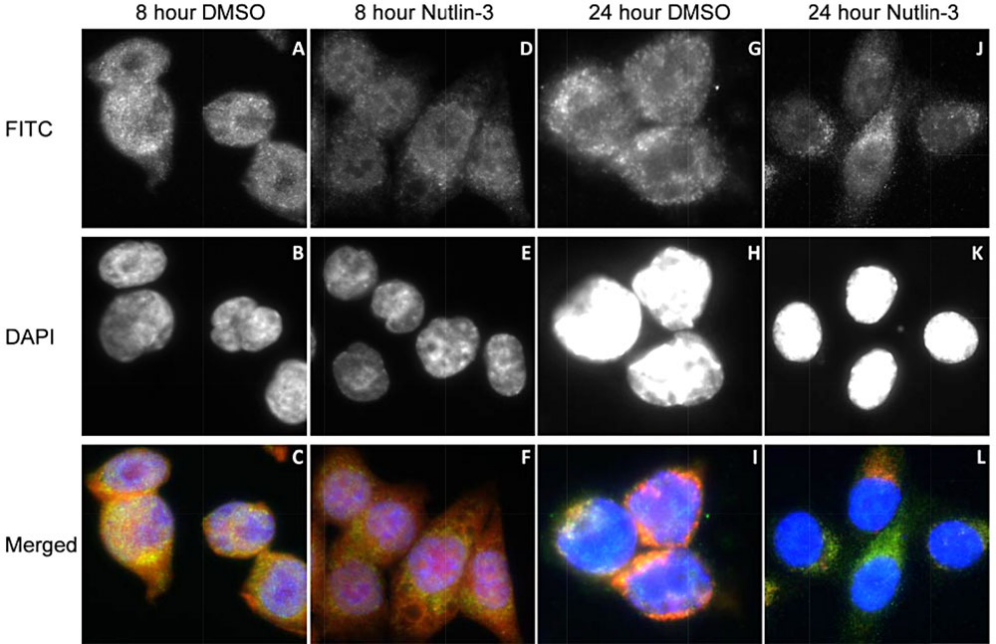
The effects of Nutlin‐3 on mitochondrial membrane permeability. HCT116 cells were treated with Nutlin‐3 for 8 or 24 h (as indicated and cells were processed using antibodies to dihydrolipoamide dehydrogenase (A, D, G, J), DAPI nuclear stain (B, E, H, K), and as a merged image (C, F, I, L). The red fluorescence highlights active mitochondria, green depicts the expression of dihydrolipoamide dehydrogenase, and degrees of colocalization as shades or orange/yellow.

As dihydrolipoamide dehydrogenase is a mitochondrial protein, we evaluated whether Nutlin‐3 might change mitochondria activity. If so, this would suggest that one dominating effect of Nutlin‐3 on cell integrity resides in perturbation of certain mitochondrial proteins. Thus, we evaluated more global effects of mitochondrial integrity using Mitotracker, a dye that binds to proteins in intact mitochondria with a functional negative membrane potential and that results in red color reflecting active mitochondria 34. At four or eight hour time points there was little change in the bioactivity of mitochondria using this assay (Fig. 4A–C versus 4D–F; 8‐h time point). By 24 h where nuclear morphology remains intact, significant loss of mitochondrial membrane potential (as defined by loss of red color) was observed in Nutlin‐3 treated cells (Fig. 4G‐I versus and 3J‐L).

**Figure 5:**
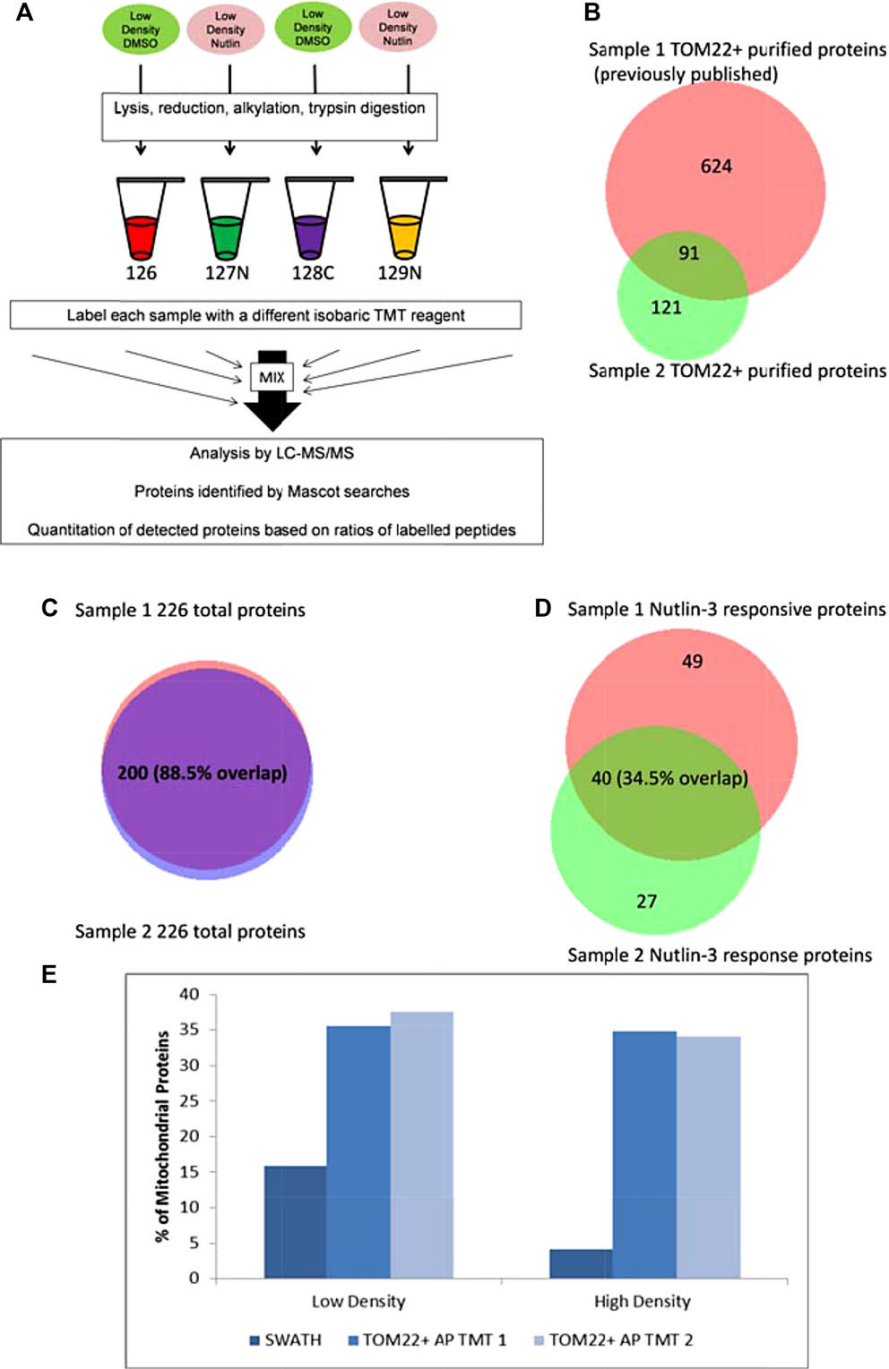
The effects of Nutlin‐3 on the proteome of TOM22+ immunoaffinity purified mitochondria. (A). The mass tags used for each sample is as indicated, without and without Nutlin‐3, done in duplicate for a total of four samples using a TMT labeling kit. (B) A comparison of the protein overlap in the TOM22 immunoprecipitate from this study (green) and a previous study (red) [37]. (C) The percentage of proteins defined to be mitochondrial using Mitominer (http://mitominer.mrc‐mbu.cam.ac.uk) in the SWATH‐MS datasets versus the TMT datasets. (D) The total protein identification (Supporting Information Tables 3 and 4) from two independent cell plates grown were compared to determine the reproducibility in protein capture. (E) Normalized Nutlin‐3 responsive changes (twofold changes) in the mitochondrial proteome (TOM22+) derived from the biological replicates summarized in Supporting Information Tables 3 and 4.

Next, we evaluated whether we could observe changes in the mitochondrial proteome four hours post‐Nutlin‐3 treatment by purifying mitochondria using antibody coupled superparamagnetic beads that bind to the TOM22 outer membrane receptor of mitochondria (Fig. 5A 35). Using this method, we performed mitochondrial immunoprecipitations in two biological replicates (at 40% cell density) to define the extent overlap of differentially responding proteins. Although this method has an advantage of speed over traditional multiple centrifugation methods of purifying mitochondrial, its limitation is that TOM22‐mitochondria are not purified. Mitochondrial samples (e.g. TOM22+ fractions) from cells treated for four hours with Nutlin‐3 were first lysed and processed using the FASP method for labeling peptides using tandem mass tag isotopic labels^36^ (Fig. 5A). A comparison of the protein identifications using the TOM22 affinity purification methodology between a prior study and this study is shown in Fig. 5B.

One thing to note is the high identity of the total protein IDs in two biological replicates (Fig. 5C and Supporting Information Tables 3 and 4). These data together suggest first that the method can reproducibly capture “stably” associated mitochondrial proteins in the immunoprecipitate and subsequent washing steps. Second, the data suggest that there are no major rearrangements of the abundant mitochondrial proteome four hours after Nutlin‐3 treatment. Nevertheless, we cannot rule out that many of these proteins are contaminants isolated through their affinity for the magnetic bead matrix, the TOM22 antibody coupled to the beads that enriches for mitochondria, or that bind mitochondrial membrane proteins specifically but artifactually after tissue lysis that disrupts subcellular organization. However, we can state that this method is an established tool that can highly enrich for TOM22+ mitochondria 35 and that a proteomic analysis of proteins isolated by this method 37 identifies many of the same proteins from our cell line (Fig. 3B).

Although the method reveals a high degree of overlap in the total protein composition using TMT (Fig. 5C), both immunoprecipitates exhibited quantitative difference in the total average fold change in response to Nutlin‐3 (Fig. 5D). For example, sample 1 showing an average difference of 13.25 and sample 2 showing an average difference of 2.04 (Supporting Information Tables 3 and 4). This identifies the variability in the methodology and suggests that the washing steps might result in differential loss of proteins that affects peptide yield in the isotopic label. Nevertheless, upon comparison of the two replicates, and upon normalization of the data in Supporting Information Tables 3 and 4, reproducible Nutlin‐3 induced changes in proteins were observed in 40 of the ∼200 proteins identified in the mitochondrial immunoprecipitation (Fig. 5D). These data are consistent with the shotgun MS data acquired using SWATH‐MS that highlighted a relatively small change in proteome changes in response to Nutlin‐3 four hours posttreatment (Fig. 2). Within the biological replicates, we stratified those proteins commonly upregulated or downregulated by 1.5‐fold differences following Nutlin‐3 treatment (Table 1). An apparent enrichment of mitochondrial proteins using the TOM22 affinity purification methodology was observed, relative to samples processed using whole cell lysis by SWATH‐MS (Fig. 5E). The data identify three mitochondrial proteins with >twofold change in expression; NADH dehydrogenase subunits are commonly upregulated in the TOM22+ mitochondrial fractions and both ADP/ATP translocase subunits are commonly downregulated in the TOM22+ mitochondrial fractions (Table 1). Additional mitochondrial proteins that show differential protein expression (from the total protein data in Supporting Information Tables 3 and 4) are highlighted (Table 1). Together the data suggest that Nutlin‐3 can impact on dynamics of the mitochondrial proteome and is consistent with the concept that Nutlin‐3 can alter mitochondrial bioactivity (Fig. 4).

### 3.4 Changes in dihydrolipoamide dehydrogenase protein–protein interaction landscape after Nutlin‐3 treatment

**Figure 6:**
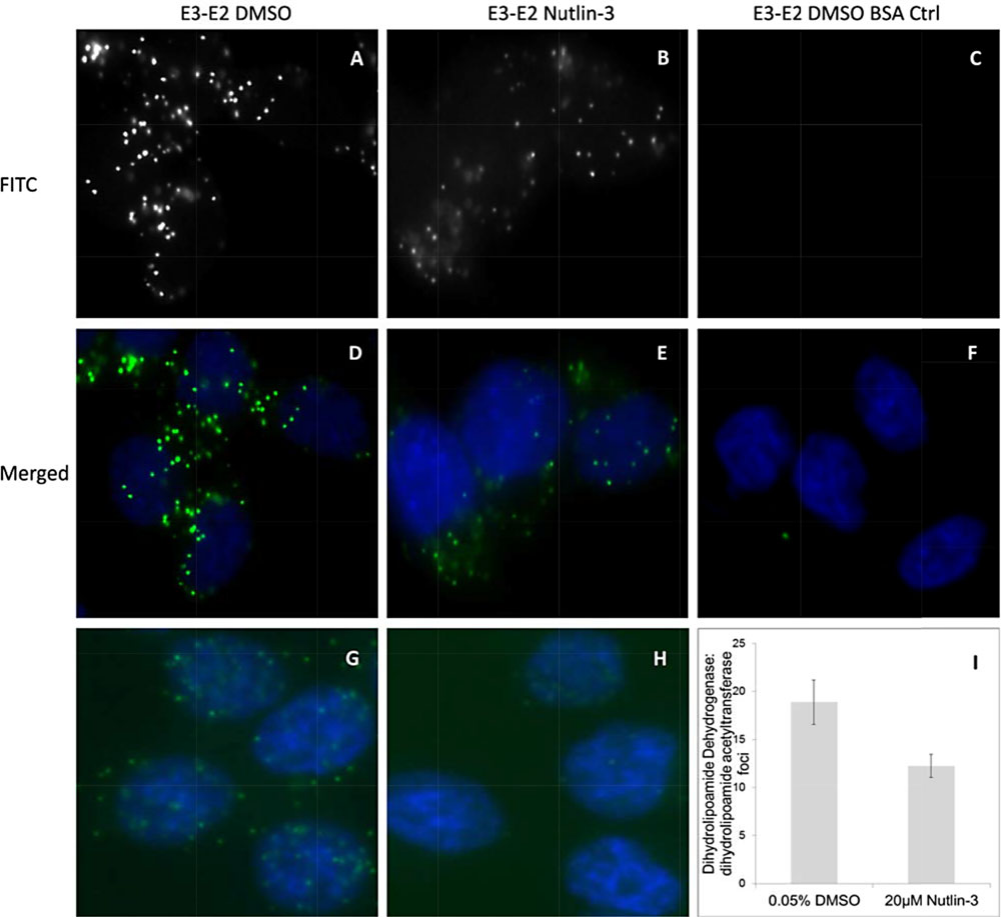
Nutlin‐3 dissociates dihydrolipoamide dehydrogenase and dihydrolipoamide acetyltransferase protein–protein interactions within the pyruvate dehydrogenase holoenzyme complex. (A–F) HCT116 cells were treated with DMSO or Nutlin‐3 (20 μM) for 4 h. Cells were fixed and processed for proximity ligation 24 as recorded in the Methods using antibodies to dihydrolipoamide dehydrogenase (mouse) and dihydrolipoamide acetyltransferase (rabbit). Cells were imaged using FITC as a readout of proximity ligation (representative images in A, B, and C), DAPI plus merged FITC (D, E, and F). (A, D) DMSO control; (B, E) Nutlin‐3 effects; (C, F) DMSO with BSA controls without primary antibodies (G and H). MDM2:p53 complexes were evaluated with DMSO control (G) or with Nutlin‐3 (H; representative images are a merge of FITC proximity ligation and DAPI to localize the nucleus). The quantitation of the average dihydrolipoamide dehydrogenase/dihydrolipoamide acetyltransferase foci in cells is summarized in I (using ImageJ software).

Showing that the mitochondrial proteome, mitochondrial activity, and dihydrolipoamide dehydrogenase change after Nutlin‐3 treatment, we next determined whether (i) MDM2 forms direct interactions with dihydrolipoamide dehydrogenase in cells, and (ii) whether dihydrolipoamide dehydrogenase protein interactions change in response to Nutlin‐3. Dihydrolipoamide dehydrogenase is the E3 Component of the three proteins that comprise the pyruvate dehydrogenase complex 38. We first examined as a positive control whether dihydrolipoamide dehydrogenase (the E3 subunit) and dihydrolipoamide acetyltransferase (the E2 subunit) form detectable protein–protein interactions in cells and whether in turn this was altered by Nutlin‐3. Proximity ligation assays were used as a tool that can identify a protein–protein interaction with a distance of 10–30 nm that is in the upper range of that observed using FRET (5–20 nm) 24, 39. This can detect authentic endogenous proteins in situ and does not rely on transfected or artificially GFP‐tagged protein vectors. Dihydrolipoamide dehydrogenase and dihydrolipoamide acetyltransferase do indeed form protein–protein interaction foci in growing cells (Fig. 6A and D). This is consistent with the known interaction between the two proteins as components of the pyruvate dehydrogenase complex. The treatment with Nutlin‐3 reduced the number of dihydrolipoamide dehydrogenase and dihydrolipoamide acetyltransferase protein–protein interaction foci (Fig. 6B and E and quantified in 6I). A negative control without primary antibodies is shown in Fig. 6C and F. As a positive control, preformed complexes of MDM2:p53 in proliferating cells (Fig. 6G) are dissociated by Nutlin‐3 (Fig. 6H). Quantitation revealed that the average number of p53:MDM2 foci per cell in control groups was 26 and after Nutlin‐3 treatment this was reduced to an average of 12 complexes per cell (data not shown). The data together suggest that the pyruvate dehydrogenase holoenzyme complex is being dissociated by Nutlin‐3 and that the integrity of the mitochondrial organelle is starting to be compromised at this relatively early time point post‐Nutlin‐3 treatment. This is consistent with a prior report of Nutlin‐3 inducing reactive oxygen species in the mitochondria and causing p53 translocation to the mitochondria 21, 32.

**Figure 7:**
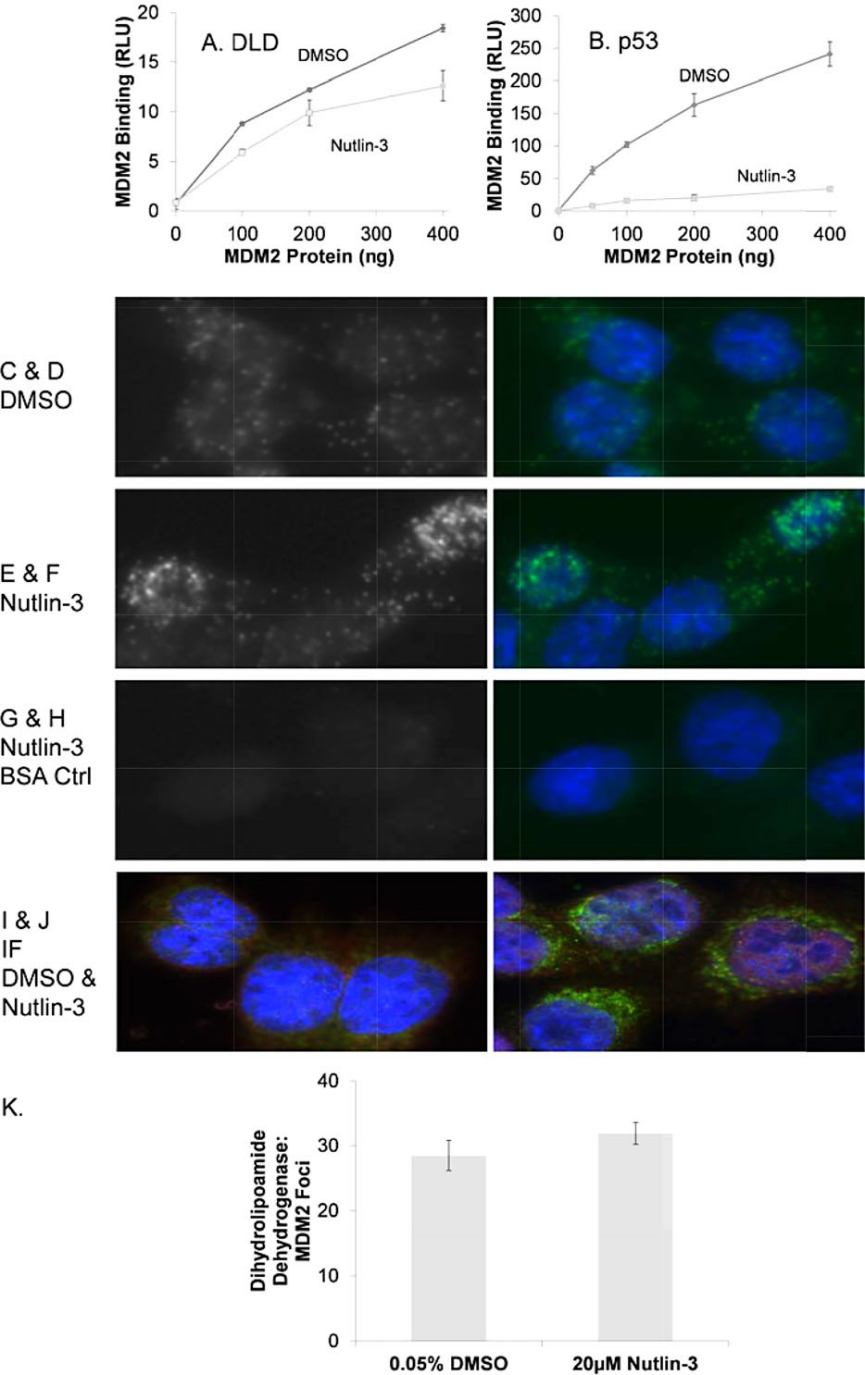
The effects of Nutlin‐3 on dihydrolipoamide dehydrogenase and MDM2 complexes protein in vitro and in vivo. (A and B) The effects of Nutlin‐3 on MDM2 protein–protein interactions. An ELISA was used to measure the binding of MDM2 to A, dihydrolipoamide dehydrogenase or B, p53 51. The purity of the indicated proteins measure by Coomassie blue is in Supporting Information Fig. 2. Target protein p53 or dihydrolipoamide dehydrogenase was coated onto the solid phase as indicated in the Methods. Ligand‐free MDM2 (DMSO control) or Nutlin‐3 (20 μM in DMSO) bound MDM2 was titrated into reactions followed by immunochemical quantitation of the amounts of MDM2 bound. MDM2 activity is depicted in relative light units as a function of increasing MDM2 protein levels (in nanograms). (C–H) In vivo binding of MDM2 and dihydrolipoamide dehydrogenase. Proximity ligation assays were used according to the Methods to measure MDM2 and dihydrolipoamide dehydrogenase binding in situ. (C and D) FITC and DAPI merged images, respectively, in proliferating cells treated with DMSO control. (E and F) FITC and DAPI merged images, respectively, in cells treated with Nutlin‐3 (20 μM) for 4 h. (G and H) FITC and DAPI merged images, respectively, in proliferating cells treated with DMSO control using BSA in place of primary antibodies as a negative control. (I and J) Total cellular distribution of MDM2 and dihydrolipoamide dehydrogenase. Immunofluorescence was used with specific antibodies in fixed cells according to the Methods to measure the total distribution of MDM2 and dihydrolipoamide dehydrogenase pools in the absence or presence of Nutlin‐3 (20 μM). (K) Quantitation of MDM2 and dihydrolipoamide dehydrogenase protein–protein interaction foci in the absence and presence of Nutlin‐3 using proximity ligation 24.

The relatively rapid change in dihydrolipoamide dehydrogenase after Nutlin‐3 treatment might lead to a change in a direct protein–protein complexes with MDM2. On the contrary, there might be no direct protein–protein interaction between dihydrolipoamide dehydrogenase and MDM2. This would indicate the effects of Nutlin‐3 on dihydrolipoamide dehydrogenase are indirect from MDM2. We next evaluated whether dihydrolipoamide dehydrogenase and MDM2 form detectable protein–protein interactions in cells and whether in turn this equilibrium is perturbed by Nutlin‐3. First, recombinant dihydrolipoamide dehydrogenase purified from bacteria was able to bind to recombinant bacterially expressed MDM2, with a marginal effect of Nutlin‐3 on the protein–protein interaction (Fig. 7A). By contrast, full‐length p53 can be dissociated more significantly by Nutlin‐3 (Fig. 7B), presumably because p53 binds to MDM2 though the N‐terminal peptide‐binding domain of MDM2. These data indicate that MDM2 can form a direct complex with dihydrolipoamide dehydrogenase but the complex is not intrinsically sensitive to Nutlin‐3.

We next determined whether endogenous MDM2 and dihydrolipoamide dehydrogenase form a protein–protein complex in cells. This was measured using the proximity ligation assay. Using this method, we observe approximately 20–30 MDM2:dihydrolipoamide dehydrogenase foci per cell (Fig. 7C and D) indicating that MDM2 and dihydrolipoamide dehydrogenase can indeed form protein–protein complexes in vivo. The number of foci is similar to that observed between MDM2 and p53 (Fig. 6) suggesting that dihydrolipoamide dehydrogenase represents a relatively dominant‐binding partner of MDM2. In response to Nutlin‐3 there is a marginal increase in the number of dihydrolipoamide dehydrogenase‐MDM2 foci in cells (Fig. 7E and F; quantified in K). However, this quantitation underestimates the qualitative change in protein‐interaction foci, as aggregates of dihydrolipoamide dehydrogenase and MDM2 form in the nucleus (Fig. 7F, arrows). Approximately 20–25% of Nutlin‐3 treated cells contain the aggregated nuclear dihydrolipoamide dehydrogenase‐MDM2 foci (data not shown). The total MDM2 and dihydrolipoamide dehydrogenase pools in the nucleus using immunofluorescence before and after Nutlin3‐treatment are shown in Fig. 5I and J. In untreated cells, the majority of dihydrolipoamide dehydrogenase and MDM2 are in the cytosol (Fig. 7I). After Nutlin‐3 treatment, the majority of MDM2 is in the nucleus and the majority of dihydrolipoamide dehydrogenase is in the cytoplasm (Fig. 7J). Thus, the apparent increase in dihydrolipoamide dehydrogenase/MDM2 aggregates in the nucleus is related to the elevated Nutlin‐3 induction of MDM2 in the nucleus.

**Figure 8:**
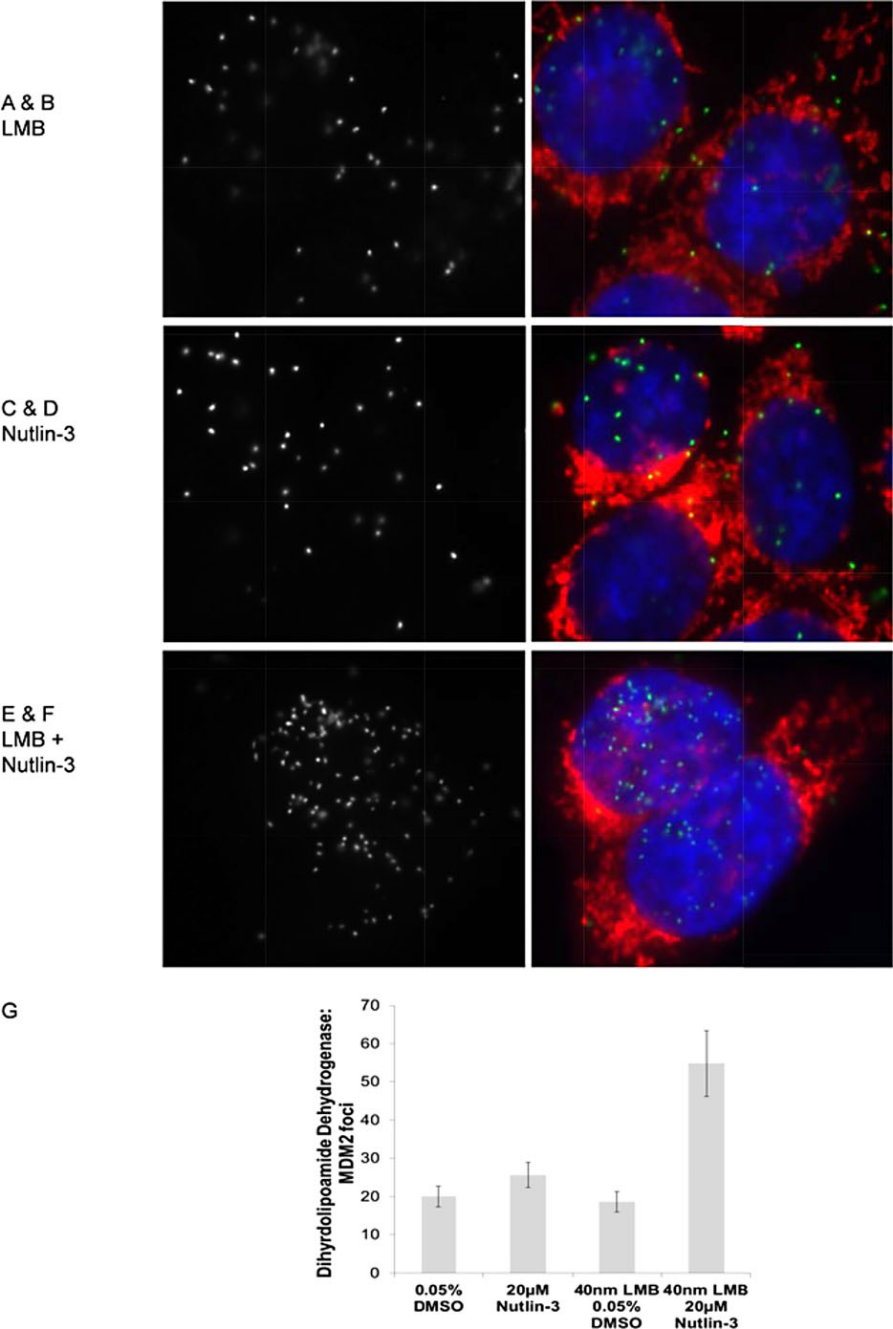
The effects of Leptomycin B on dihydrolipoamide dehydrogenase and MDM2 complexes in cells using proximity ligation assays. (A and B) The effects of Leptomycin B after four hours of treatment on MDM2 and dihydrolipoamide dehydrogenase protein–protein interactions with images depicting FITC proximity ligation [24] (A) and DAPI nuclear stain (in blue) and MitoTracker Red CMXros (in red) as a merged image (B). (C and D) The effects of Nutlin‐3 on MDM2 and dihydrolipoamide dehydrogenase protein–protein interactions with images depicting FITC proximity ligation (C) and DAPI nuclear stain (in blue) and MitoTracker Red CMXros (in red) as a merged image (D). (E and F) The effects of Leptomycin B and Nutlin‐3 combined on MDM2 and dihydrolipoamide dehydrogenase protein–protein interactions with images depicting FITC proximity ligation (E) and DAPI nuclear stain (in blue) and MitoTracker Red CMXros (in red) as a merged image (F). (G) Quantitation of protein–protein interaction foci with the indicated treatment with DMSO, Leptomycin B, Nutlin‐3, or Nutlin‐3, and Leptomycin B combined.

In order to examine whether the nuclear pools of dihydrolipoamide dehydrogenase and MDM2 in the nucleus are dynamic, we compared the effects of the nuclear export inhibitor Leptomycin B on Nutlin‐induced dihydrolipoamide dehydrogenase‐MDM2 nuclear foci using proximity ligation assays. Compared to Nutlin‐3 treatment (Fig. 8A and B) or Leptomycin B treatment only (Fig. 8C and D), significantly more MDM2:dihydrolipoamide dehydrogenase foci were seen in the nucleus with combined treatment of Leptomycin B and Nutlin‐3 (Fig. 8E and F, quantified in G).

## 4 Discussion

MDM2 is a target for drug‐discovery programmes aiming to engage the p53 anti‐tumor response. Recent clinical trials evaluating an MDM2 inhibitor (RG7112; a member of the Nutlin family) in sarcoma patients gave encouraging results with maintenance of stable disease [8]. Thus, innovative approaches that convert the clinical response from stable disease to reduced tumor volume could establish a key proof‐of‐concept for drugging MDM2 in human cancer. The Nutlin class of MDM2 drugs activates p53 transcription function but these drugs do not inhibit MDM2 E3‐ligase activity 16. In fact, Nutlin has a partial agonist effect and can stabilize prooncogenic components of the MDM2 protein–protein interaction landscape such as Notch 18 (Fig. 1). Our hypothesis is that the balance between the MDM2 mediated prooncogenic and proapoptotic pathways engaged by the agonist function of Nutlin's impacts on therapeutic outcome 11. This will likely be cell (patient/cancer genome) specific and influenced by the cellular environment, especially as cell density or cell number can impact on MDM2 levels 19 and heterogeneity in p53 protein levels in cancers in vivo 29.

This cell density effect on MDM2 protein levels (Fig. 1; 19) complicates, methodologically, identifying “core” MDM2 interacting proteins. For instance, a key study has previously highlighted the ability of MDM2 to promote p53 protein synthesis 28 in addition to the classically known role of MDM2 to catalyze p53 protein degradation 4. Importantly, this former data suggested that confluence‐dependent signaling pathways regulate MDM2‐mediated synthesis or degradation of p53, with lower density promoting higher levels of MDM2 that changes the ratio of p53 synthesis to p53 protein degradation 28. These data suggest that other MDM2‐binding proteins could also be subjected to density effects on MDM2 signaling. As such a key study design we used was to incorporate cell density changes into the proteome screens aimed to define the “core” MDM2 responsive proteins. A second key study design was to focus on defining proteome changes at a relatively early time point (4 h post‐Nutlin‐3 treatment) to identify proteome changes more likely to respond to primary impacts on MDM2 ligand binding before the growth arrest or apoptotic machinery is activated. Lastly, we have also used a label‐free method for quantitative proteomics (SWATH) that provided us with a deeper coverage of differential changes in the steady‐state proteome upon Nutlin‐3 treatment (Supporting Information Tables 1 and 2) than we had previously obtained using isobaric data‐dependent labeling methods 11.

By combining the SWATH acquired dataset at both densities where MDM2 protein levels are different (Fig. 2A and B), we can start to identify cell‐density independent targets in the MDM2 pathway analysis. The experimental approach we used did not incorporate biological replicates at two cell densities that would be required to produce statistically validated pathway maps. Nevertheless, the approach allowed us to identify dihydrolipoamide dehydrogenase as an “outlier” at the two densities where MDM2 protein levels differ (Fig. 1) and to subsequently validate its rearrangement with MDM2 in drug‐treated cells. The mechanism whereby MDM2 protein levels and p53 pathway change as a function of cell density is only beginning to be defined. Phosphorylation of the MDM2 pseudo‐substrate motif can reduce is steady‐state levels and simultaneously reduce p53 protein levels 19. The kinase‐phosphatase axis that targets this MDM2 motif as a function of high cell density is not defined. In addition, the reduction in p53 activity at high cell density has been linked to change in cell–cell junctions, not stage of cell cycle 20. The changes in cell–cell junctions at high density whereby p53 activity is suppressed might more accurately reflect intratumor heterogeneity in vivo, where p53 protein levels show significant heterogeneity 29. It remains to be determined whether the partial resistance of human cancers to MDM2 drugs in clinical trials 8 is linked to density effects on MDM2 functions. Dissecting this effect in vivo might be possible in future as is has been shown that different metabolic zones with distinct proteomic signatures exist in different regions of the tumor 40. The application of existing protocols to process microdissected formalin fixed clinical samples for proteomics screens 41 might define such tumor heterogeneity to impact on MDM2 drug biomarker discovery.

Using this strategy, we show that only two proteins can be identified as changing greater than 2 x log_2_ as a function of cell density; the mitochondrial protein dihydrolipoamide dehydrogenase and the nuclear protein LRWD1 (Fig. 2A and B). These targets provide clues into two possibly dominant cellular pathways that might be most affected by MDM2 drugs independent of cell‐cycle stage or cell density. In particular the focus on the early time point of 4 h to capture primary proteome changes under conditions in which p53:MDM2 complexes are reduced, we hoped to obtain new insight into dominant biological processes of MDM2. LRWD1 is reported to be a methylation‐sensitive nucleosome interactor that is recruited by histone methylation 31. Although LRWD1 is also reported to be a centrosomal protein 42, its major effect appears to be as a replication origin recognition complex‐associated (ORCA/LRWD1), that binds to methylated H3K9 targets and interacts with G9a/GLP and Suv39H1 in a chromatin context‐dependent manner 43. It will be interesting to define the mechanism whereby MDM2 might regulate the LRWD1/ORCA axis under normal conditions and how Nutlin‐3 impacts upon this biological pathway.

Interestingly, many of the differentially expressed Nutlin‐3 responsive proteins showed inverse changes at the low or high densities. LRWD1 showed increases at both cell densities; Mitochondrial acyl‐coenzyme A thioesterase 9 ACOT9 was upregulated at 40% cell density and suppressed at 80% cell density; while RNA‐binding motif protein 7 isoform RBM7 was suppressed at low 40% cell density and elevated at 80% cell density (Fig. 2B). This apparent variation could reflect true changes in MDM2 protein levels 19 and pathway signaling as a function of cell density. For example, p53 protein is stabilized at either density by Nutlin‐3 (Fig. 1), but MDM2 is only stabilized at the lower density (as in Fig. 1). Alternatively, it could reflect true variability on the kinetics of induction in different cell populations of protein changes since the cells were harvested four hours posttreatment to capture primary responders. Most notably, dihydrolipoamide dehydrogenase exhibited large fold changes but differential induction or suppression as a function of the two cell densities.

**Figure 9:**
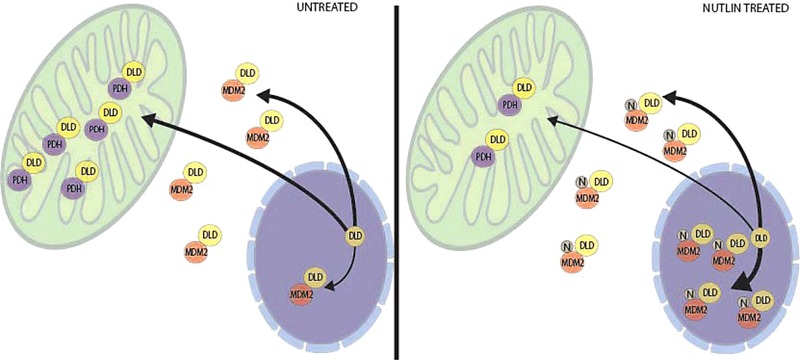
Model summarizing the effects of Nutlin‐3 on dihydrolipoamide dehydrogenase and MDM2 localizations. (Left panel) In proliferating cells, there are at least two pools of dihydrolipoamide dehydrogenase. One pool interacts predominantly with components of the pyruvate dehydrogenase complex and the second pool with MDM2, predominantly in the cytosol. (Right panel) Following Nutlin‐3 treatment, perturbation of the mitochondrial proteome results in dissociation of the dihydrolipoamide dehydrogenase/dihydrolipoamide acetyltransferase, suggesting a disruption of pyruvate dehydrogenase holoenzyme complex. In addition, although minimal dihydrolipoamide dehydrogenase is observed in the nucleus in untreated cells, the Nutlin‐3‐dependent import of MDM2 into the nucleus coincides with increased dihydrolipoamide dehydrogenase: MDM2 complexes in the nucleus. It remains to be determined if the binding of dihydrolipoamide dehydrogenase to MDM2 in the nucleus has a direct effect on p53 protein activation.

In our current manuscript, we focused our validation on the mitochondrial protein dihydrolipoamide dehydrogenase, as suggestive evidence that MDM2 impacts on mitochondrial proteome might be an early target of Nutlin‐3 effects. This was due to the prior evidence that p53 translocation into the mitochondria is linked to Nutlin‐induced p53‐mediated apoptosis 32. We identified dihydrolipoamide dehydrogenase as a novel MDM2‐binding protein as judged by in vitro and in vivo cobinding assays and linked this Nutlin‐3 induced change to a decrease in the integrity of the pyruvate dehydrogenase complex. This adds to our growing knowledge of how Nutlin‐3 effects the mitochondria. It not only causes p53 protein translocation, but directly effects a specific MDM2 protein–protein interaction with dihydrolipoamide dehydrogenase with an early dissociation of the pyruvate dehydrogenase holoenzyme complex (Fig. 9). Understanding further the role of Nutlin‐3 induced dihydrolipoamide dehydrogenase:MDM2 complexes in the nucleus (Fig. 9) might shed new light on how dihydrolipoamide dehydrogenase might control p53 activation in the nucleus or regulate other MDM2 protein interactions in the nucleus, such as LRWD1.

In order to further evaluate the effects of cell density on the mitochondrial proteome itself, we utilized a mitochondrial affinity purification method that captures TOM22, a mitochondrial outer membrane receptor 44. The reproducibility in total mitochondrial proteins identified using the mitochondrial immunoprecipitation coupled to TMT is relatively high using the two biological replicates. However, with an obvious variability in the fold changes in the common mitochondrial proteome (as defined by TOM22+ enriched material; Supporting Information Tables 3 and 4), there were proteins commonly altered in the two biological replicates. Proteins that are affinity purified using this method are not only classically known mitochondrial proteins, but additional targets such as histones (Table 1). Although this might suggest that the affinity purification method artifactually captures free histones, there are prior reports on the effects of free histones on mitochondrial organelle integrity 45, 46, 47.

It is also important to point out that there is no comprehensive overlap in the proteome data using SWATH and mitochondrial‐TMT methods that define Nutlin‐3 responsive proteins. The SWATH method was applied to ask what abundant total cellular proteins changes occur in response to Nutlin‐3. The Mitochondrial‐TMT was applied to ask what TOM22+ mitochondrial proteins change in response to Nutlin‐3. Technically, an explanation for this difference could be due in part to the buffers used in lysis coupled to the methods applied. For example, SWATH uses urea lysis buffer that denatures all proteins from a cell pellet and can thus capture the vast majority of cellular proteins whether soluble or insoluble. By contrast, the mitochondrial isolation uses soluble lysis buffers, which maintains native conformations, and leaves behind significant insoluble proteins in the pellet after lysis and centrifugal clarification. Proteases can also function under these native immunoprecipitation conditions and the time required for mitochondrial enrichment can result in loss of some mitochondrial binding proteins. Thus, both methods can be considered complimentary approaches to ask distinct questions. Generally, the SWATH can be applied to ask what abundant proteins changes in response to Nutlin‐3. The Mitochondrial‐TMT is applied to ask what TOM22+ mitochondrial proteins change in response to Nutlin‐3. Lastly, although we can identify proteins that are up or downregulated by Nutlin‐3 (or neutral) in the TOM22 affinity purification, it is important to keep in mind that TOM22 is a receptor for the apoptotic protein Bax 48. If Nutlin‐3 impacts early on Bax release, then this might alter the TOM22 receptor to impact on the drug‐induced changes. Nevertheless, altogether, our data begin to provide protocols to define specific mitochondrial biomarkers of MDM2 drug leads that might be useful to design new combination drug leads or provide new biomarkers to measure effects of MDM2 targeted drugs in vivo.

In conclusion MDM2 is an E3 ubiquitin ligase that functions in proteostasis to control p53 function. MDM2 drugs are just emerging as compelling agents to treat wt‐p53 cancers that have the *mdm2* gene amplified [8]. The current class of drugs target the N‐terminal allosteric peptide‐binding pocket in MDM2. However, these molecules do not inhibit the MDM2 ubiquitin ligase function but they can activate or inhibit MDM2 protein–protein interactions. In cell culture, this can translate into data showing that Nutlin‐3 can stabilize p53 protein or Notch proteins [10]. The differential induction of tumor suppressor (p53) or oncogenic proteins (Notch) might regulate the balance of cancer cell death or survival. This “oncogenic” biomarker signature of Nutlin‐3 might be one reason why patients tumors do not regress after drug treatment despite the biomarker p53 pathway being “activated” [8]. Thus, developing proteomic biomarker screens to identify “dominant” MDM2 drug responsive proteins forms an important platform for future patient stratification. In this report, we use SWATH‐MS to identify dominant Nutlin‐3 responsive proteins as a concept screen that can be used to identify possibly important MDM2 interacting proteins. Incorporated into the screen is the concept that the MDM2 interactome can change as a function of cell density and that this builds‐in “MDM2 heterogeneity” into the biomarker discovery process. We validate one of the few commonly perturbed proteins, dihydrolipoamide dehydrogenase, a subunit of the mitochondrial pyruvate dehydrogenase complex. We also use orthogonal assays to evaluate Nutlin‐3 effects on the mitochondria including (i) mitotracker that defines mitochondrial membrane potential; (ii) TMT screens of affinity purified mitochondrial to determine key mitochondrial associated proteins that changed early after drug treatment; and (iii) proximity ligation assays to demonstrate that dihydrolipoamide dehydrogenase protein–protein interactions are rearranged in cells. Altogether, this platform provides a roadmap that can be applied to clinical samples to begin to stratify MDM2 drug treated patients and begin to link tumor responses to MDM2 interacting biomarkers.

*The authors have declared no conflict of interest*.

## Supporting information

As a service to our authors and readers, this journal provides supporting information supplied by the authors. Such materials are peer reviewed and may be re‐organized for online delivery, but are not copy‐edited or typeset. Technical support issues arising from supporting information (other than missing files) should be addressed to the authors.

Supplementary Figure 1. Example of chromatographic data acquired to validate SWATH reproducibility in technical replicates. A shows total ion current (TIC) from 3 technical replicates of Nutlin‐3 p53+ sample from 40% cell density. It highlights the accuracy of the autosampler sample pickup and reproducibility of sample loading. From the summary it is evident that one replicate (pink) has a slightly lower TIC intensity. Such intensity deviations are corrected by normalisation on total ion current in Markerview software. Markerview software frequently operates with peak areas extracted from a sample pair to be compared and to provide unbiased sample comparison it is necessary to normalise TICs of technical replicates from both samples (usually 6 measurements when comparing a samples pair) at once. B shows an extracted ion chromatogram of random mass (m/z 851.4) from three technical replicates. This gives insight on retention time reproducibility that is important for determining the correct retention time extraction window using the Peakview software. Retention time window describes the LC retention time shifts between SWATH sample technical replicates and DDA measurement and specifies in which scope of retention times software should look for peaks included in spectral library. XICs suggest that retention times were quite stable and we allowed narrower retention time extraction windows (3.5 min; see Materials and Methods) that improved peak picking and protein quantitation.
Supplementary Figure 2. The Coomassie blue stained SDS gels show the relative purity and molecular mass of the recombinant full length MDM2, p53, and DLD used in the ELISA (Figure 7).

Suppl. Table S1

Suppl. Table S2

Suppl. Table S3

Suppl. Table S4
