# Supplementary material for: Rearrangement of mitochondrial pyruvate dehydrogenase subunit dihydrolipoamide dehydrogenase protein–protein interactions by the MDM2 ligand nutlin‐3
Source: Proteomics. 2016 Sep 5;16(17):2327–44. doi: 10.1002/pmic.201500501 (PMC5026170; doi:10.1002/pmic.201500501)
Supplement: Supplementary file 1 — Supplementary Figure 1. Example of chromatographic data acquired to validate SWATH reproducibility in technical replicates. A shows total ion current (TIC) from 3 technical replicates of Nutlin‐3 p53+ sample from 40% cell density. It highlights the accuracy of the autosampler sample pickup and reproducibility of sample loading. From the summary it is evident that one replicate (pink) has a slightly lower TIC intensity. Such intensity deviations are corrected by normalisation on total ion current in Markerview software. Markerview software frequently operates with peak areas extracted from a sample pair to be compared and to provide unbiased sample comparison it is necessary to normalise TICs of technical replicates from both samples (usually 6 measurements when comparing a samples pair) at once. B shows an extracted ion chromatogram of random mass (m/z 851.4) from three technical replicates. This gives insight on retention time reproducibility that is important for determining the correct retention time extraction window using the Peakview software. Retention time window describes the LC retention time shifts between SWATH sample technical replicates and DDA measurement and specifies in which scope of retention times software should look for peaks included in spectral library. XICs suggest that retention times were quite stable and we allowed narrower retention time extraction windows (3.5 min; see Materials and Methods) that improved peak picking and protein quantitation. Supplementary Figure 2. The Coomassie blue stained SDS gels show the relative purity and molecular mass of the recombinant full length MDM2, p53, and DLD used in the ELISA (Figure 7). [file PMIC-16-2327-s001.docx]

**Supplementary Figure 1.** Example of chromatographic data acquired to validate SWATH reproducibility in technical replicates. A shows total ion current (TIC) from 3 technical replicates of Nutlin-3 p53+ sample from 40% cell density. It highlights the accuracy of the autosampler sample pickup and reproducibility of sample loading. From the summary it is evident that one replicate (pink) has a slightly lower TIC intensity. Such intensity deviations are corrected by normalisation on total ion current in Markerview software. Markerview software frequently operates with peak areas extracted from a sample pair to be compared and to provide unbiased sample comparison it is necessary to normalise TICs of technical replicates from both samples (usually 6 measurements when comparing a samples pair) at once. B shows an extracted ion chromatogram of random mass (m/z 851.4) from three technical replicates. This gives insight on retention time reproducibility that is important for determining the correct retention time extraction window using the Peakview software. Retention time window describes the LC retention time shifts between SWATH sample technical replicates and DDA measurement and specifies in which scope of retention times software should look for peaks included in spectral library. XICs suggest that retention times were quite stable and we allowed narrower retention time extraction windows (3.5 min; see Materials and Methods) that improved peak picking and protein quantitation.


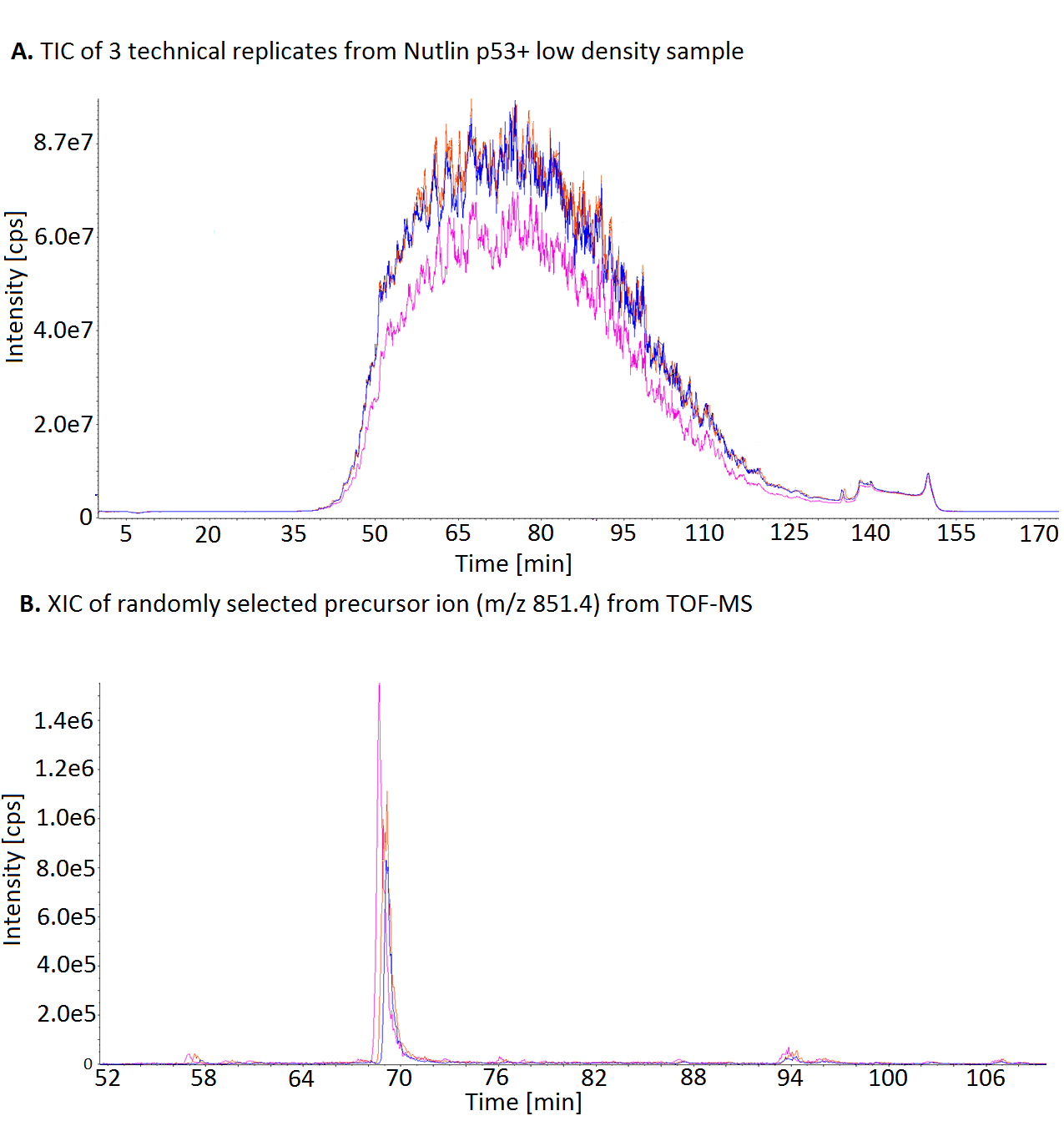


Supplementary Figure 2: The Coomassie blue stained SDS gels show the relative purity and molecular mass of the recombinant full length MDM2, p53, and DLD used in the ELISA (Figure 7).
